# SARS-CoV-2 Infections in Italian Schools: Preliminary Findings After 1 Month of School Opening During the Second Wave of the Pandemic

**DOI:** 10.3389/fped.2020.615894

**Published:** 2021-01-14

**Authors:** Danilo Buonsenso, Cristina De Rose, Rossana Moroni, Piero Valentini

**Affiliations:** ^1^Department of Woman and Child Health and Public Health, Fondazione Policlinico Universitario A. Gemelli, Rome, Italy; ^2^Dipartimento di Scienze Biotecnologiche di Base, Cliniche Intensivologiche e Perioperatorie, Università Cattolica del Sacro Cuore, Rome, Italy; ^3^Global Health Research Institute, Istituto di Igiene, Università Cattolica del Sacro Cuore, Rome, Italy; ^4^Direzione Scientifica, Fondazione Policlinico Universitario A. Gemelli IRCCS, Rome, Italy

**Keywords:** SARS-CoV-2, COVID-19, school, students, Italy

## Abstract

**Introduction:** The impact of school opening on the severe acute respiratory syndrome coronavirus 2 (SARS-CoV-2) pandemic is still unknown. This study aims to provide preliminary information about the number of SARS-CoV-2 cases among students attending Italian schools.

**Methods:** Data are extracted and analyzed from an open-access, online dataset that monitor, on a daily basis, media news about SARS-CoV-2 infections of students attending Italian schools.

**Results:** As of October 5, 2020, a total of 1,350 cases of SARS-CoV-2 infections have been registered in the Italian territory schools (involving 1,059 students, 145 teachers, and 146 other school members), for a total of 1,212 out of 65,104 (1.8%) Italian schools involved. National schools reported only one case of SARS-CoV-2 infection in more than 90% of cases, and only in one high school a cluster of more than 10 cases has been described (*P* = 0.015). The detection of one or more SARS-CoV-2 infections led to the closure of 192 (15.8%) entire schools, more frequently nursery/kindergartens (*P* < 0.0005).

**Discussion:** Our preliminary data support low transmission of SARS-CoV-2 within schools, at least among younger students. However, entire schools are frequently closed in the fear of larger outbreaks. Continuous monitoring of school settings, hopefully through daily updated open-access datasets, is needed to better understand the impact of schools on the pandemic and provide guidelines that better consider different risks within different age groups.

## Introduction

During the first months of the COVID-19 (coronavirus disease 2019) pandemic, starting from April 2020, aiming to reduce the severe acute respiratory syndrome coronavirus 2 (SARS-CoV-2) transmission, most governments declared school closure with ~91% of the world's students in more than 190 countries confined at home (1). This has caused immeasurable disruption to the lives, learning, and well-being of children around the world. An entire generation has transiently seen its education interrupted (2).

European countries have been able to achieve a good control of the pandemic during summer. Because of the heavy consequences of the restrictive measures on the national economies, restrictive measures have been weakened, and most social activities of any type, comprehensively, opened, although masking, hand hygiene, and physical distancing were always a necessary obligation according to governments. However, since September, the number of SARS-CoV-2 cases has been progressively increasing in Europe, with countries, such as France and Spain heavily involved. Italy, the first heavily involved country after China, was one of the countries that best managed the virus during the first wave and successfully lowered cases, particularly during early summer. However, since the first days of September, Italy is back to more than 2,000 new cases per day with an increase in intensive care unit admissions and deaths. This scenario suggests that the second wave has begun in Europe. In this context, “the era of open schools” during the COVID-19 pandemic has started. One of the most important unanswered question is related to school and COVID-19. Will the school reopening have an impact on SARS-CoV-2 transmission among students and the whole community? To date, there are no evidences available to understand the impact of national school opening during the second wave of the pandemic and national governments did not provide yet official data. This information is fundamental to know how many cases within schools have been notified and if a specific group of students or type of school is more susceptible to SARS-CoV-2 clusters. A better knowledge of SARS-CoV-2 spread among students would improve the school organization during the COVID-19 pandemic, allowing making decisions that better balance benefits and risking for children and their families.

This study aims to provide preliminary information about the number of SARS-CoV-2 cases among students attending Italian schools and to assess if the type of school (and therefore age groups) is associated with different rates of infection.

## Methods

### Study Population

On September 14, 2020, two Ph.D. students developed an open-access, online dataset aiming to monitor, on a daily basis, media news about SARS-CoV-2 infections of students attending Italian schools (https://bit.ly/covid_scuole; https://www.datawrapper.de/_/DLy9C). At the time of development of this dataset (September 14, 2020), no similar data were publicly available in Italy. Although the Ministry of Education requested schools to communicate this information on September 25, 2020, at the date of writing of this manuscript (October 9, 2020), there are no publicly available data about SARS-CoV-2 infections among Italian schools.

The news of cases of SARS-CoV-2 infections are selected from three main resources:

- Google News: the keyword “positive school” is searched. The resulting news is read to select only the relevant ones. For example, the news “School, a positive start” is not considered relevant.
- Institutional sites: Some institutional sites of the Italian regions provide information on the situation of SARS-CoV-2 infections within schools.
- Reports: Users send news reports through private messages or directly fill the report form. The news reported are read to verify their relevance. If a report is not accompanied by a link that testifies the credibility of the website, it is not inserted until news published by a newspaper or institution are received.

Particular attention is given to news published on the web: national and local newspapers are considered reliable sources, whereas private blogs are not. All the news are read in their entirety, to extract the variables of interest described below.

Only SARS-CoV-2 cases confirmed with a molecular (polymerase chain reaction) nasopharyngeal swab were included in the analyses, because at the time of this study the rapid antigenic nasopharyngeal tests were not yet available in Italy.

The following variables are collected within the dataset: day, region, province, city, school name, type of school, national school code, number of SARS-CoV-2–positive people within the school, category of the positive person (student, teacher, other), type of quarantine activated after a positive case is notified (the whole class, the whole school, students, teachers), school closure (yes, no), number of contacts of the index case resulted positive during epidemiologic assessment, and reference of the news.

Being the information collected from news media channels, neither ethic committee was requested, nor patients/guardian approvals needed.

### The Italian Context

In Italy, there are ~65,104 schools, including nursery schools, kindergartens, primary schools (elementary), lower secondary schools (middle), and upper secondary schools (high schools), including also the private schools (https://www.tuttitalia.it/scuole). According to the *Focus* published by the Ministry of Education, with the main data relating to the recently started 2019–2020 school year, Italian students are now more than 8 million (7,599,259 students attending national schools, 866,805 attending of peer institutions) (https://www.miuristruzione.it/13007-quanti-sono-gli-studenti-in-italia-ecco-i-dati-aggiornati-al-2019-2020/).

The Italian guidelines (3) concerning school opening during the pandemic include reduced number of students per class, or if this is not feasible, guarantee of a distance of at least 1 m between each student, frequent hand hygiene, and masking. If a child is diagnosed with SARS-CoV-2 infection, the class is quarantined for 2 weeks, but the rest of the school is requested to continue normal school activities.

### Statistical Methods

Variables present in the database were analyzed through descriptive statistical techniques. In particular, the quantitative variables are represented through the following measures: mean and standard deviation. The qualitative variables are represented with absolute frequencies and percentage. The comparison between groups was made with χ^2^.

## Results

As of October 5, 2020, a total of 1,350 cases of SARS-CoV-2 infections have been registered in the Italian territory schools (involving 1,059 students, 145 teachers, and 146 other school members), for a total of 1,212 out of 65,104 (1.8%) Italian schools involved. The distribution of cases of infections according to school is as follows: 236 (17.5%) pupils from nursery/kindergartens, 300 (22.2%) pupils from elementary schools, 208 (15.4%) pupils from middle school, 452 (33.5%) pupils from high schools, and 55 (4.1%) from peer institutions [for 99 children (7.3%), school type was not available].

Students represent 78.4% of cases. Table 1 shows that the distribution of the categories of infected people is significantly different in the different levels of schools (*P* < 0.0005).

**Table 1:** Distribution of the categories of infected people.

		**Teachers**	**Students**	**Others**	**Missing**	**Total**
Nursery/kindergarten	*n*	30	171	16	19	236
	%	12.7	72.5	6.8	8.1	100.0
Elementary	*n*	45	230	7	18	300
	%	15.0	76.7	2.3	6.0	100.0
Middle	*n*	20	177	1	10	208
	%	9.6	85.1	0.5	4.8	100.0
High school	*n*	25	384	16	27	452
	%	5.5	85.0	3.5	6.0	100.0
Peer school	*n*	11	28	7	9	55
	%	20.0	50.9	12.7	16.4	100.0
Missing	*n*	14	69	5	11	99
	%	14.1	69.7	5.1	11.1	100.0
Total	*n*	145	1,059	52	94	1,350
	%	10.7	78.4	3.9	7.0	100.0

With the exception of peers schools, national schools reported only one case of SARS-CoV-2 infection in more than 90% of cases, and only in one high school a cluster of more than 10 cases has been described (*P* = 0.015, Table 2).

**Table 2:** Number of detected cases according to school degree.

		**1 case**	**2–5 cases**	**6–10 cases**	**>10 cases**	**Details missing**	**Total**
Nursery/kindergarten	*n*	218	10	0	0	8	236
	%	92.4	4.2	0.0	0.0	3.3	100.0
Elementary	*n*	280	17	2	0	1	300
	%	93.3	5.7	0.7	0.0	0.3	100.0
Middle	*n*	198	5	1	0	4	208
	%	95.2	2.4	0.5	0.0	2.0	100.0
High school	*n*	419	25	2	1	5	452
	%	92.7	5.5	0.4	0.2	1.1	100.0
Peer school	*n*	47	3	0	0	5	55
	%	85.5	5.5	0.0	0.0	9.1	100.0
Missing	*n*	90	4	0	0	5	99
	%	90.9	4.0	0.0	0.0	5.0	100.0
Total	*n*	1,252	64	5	1	28	1,350
	%	92.7	4.7	0.4	0.1	2.0	100.0

The detection of one or more SARS-CoV-2 infections led to the closure of 192 (15.8%) entire schools. In particular, school closures were distributed as follows according to school type (calculated on the total number of each type with at least eon infection reported): 51 out of 236 nursery/kindergartens (21.6%), 38 out of 300 elementary schools (12.7%), 23 out of 208 middle schools (11.1%), 49 out of 452 high schools (10.8%), and 21 out of 55 peer schools (38.1%) (*P* < 0.0005).

## Discussion

Our preliminary study shows that, as of October 5, ~1,059 students of different age groups have been notified with SARS-CoV-2 infection. Importantly, national schools reported only 1 infection in more than 90% of cases, and only in one high school a cluster of more than 10 cases has been described (*P* = 0.015). These findings would suggest that, when proper preventive measures are respected, the intraclass transmission of the virus is low. This is in agreement with recent studies suggesting that children have lower susceptibility to SARS-CoV-2, compared with adults (4). A systematic review and misanalysis have brought to light stratification of susceptibility of COVID-19 infection of children younger than 14 years than adults, with adolescents appearing to have similar susceptibility adult. Few data are available on the onward transmission of SARS-CoV-2 from children to others. Data from a large Australian school contact-tracing study (5) suggest that, at a population level, children and adolescents might play only a limited role in the transmission of this virus. This is consistent with the data on susceptibility, suggesting that lower rates of secondary infection mean that children and adolescents have less opportunity for onward transmission. This is consistent with a national South Korean study (6), which found the secondary attack rate from children to household members was extremely low. The available studies suggest children and adolescents play a lesser role in transmission of SARS-CoV-2, which is in marked contrast to influenza (7).

Importantly, despite evidence of a low transmission of SARS-CoV-2 infection among students and our preliminary findings showing that in most cases only one child has been infected in each school, the detection of one or more infections led to the closure of 192 (15.8%) entire schools. This is in contrast with Italian guidelines (3) and even with the Centers for Disease Control and Prevention operative modalities on the return to school, which suggest quarantine for 14 days for all asymptomatic contacts of a suspicious or positive case (8). School closures in case of small clusters have potential consequences on children and families and, indirectly, on the whole of society. Activating the quarantine for all doubtful or confirmed cases means that we all expect that school continuity is not guaranteed. This has direct consequences on children's right to education and children's mental health (2), but also on their families that, having to care of quarantined children, who cannot guarantee work continuity. Indirectly, this has obvious consequences on the whole society.

If school closure should not be applied in case of small cluster, does the preventive or routine quarantine for children attending the same class of a child with SARS-CoV-2 infection have any evidence? Already at 9 months of the pandemic, we have enough evidence that ~1% of all COVID-19 cases involve children (9), that morbidity and mortality are extremely low in this age group (10), and that children play a limited role in viral spread. Children are not COVID-19 super spreaders (11).

Although the data we showed are preliminary, they may confirm that masking, hand hygiene, and physical distancing applied in school settings may limit the virus spreading. On the opposite side, school or class closures in case of isolated SARS-CoV-2 cases may be a drastic measure that brings low advantages in terms of virus control but fuels an already common sense of precariousness. However, it is important to clarify that as children are most of the times asymptomatic or pauci-symptomatic when they are infected, the real burden of cases detected at school may be underestimated. To date, while we have some studies on household patterns of SARS-CoV-2 infections (12), they were mostly made during the first wave when almost all schools were closed.

Rather than seeing schools as facilitators of the COVID-19 pandemic, we should look at schools as educational settings with a potential of supporting the fight against the pandemic. The fact that children and adolescents are requested to respect certain rules during school hours may trigger positive attitudes even outside school. This is especially important for adolescents, who are in need of social relationships but bear a potentially higher susceptibility to SARS-CoV-2 (4).

Considering the heavy indirect consequences of early lockdowns, the social and scientific communities are aware that we must find the proper balance between a near-normal life and the best control of the pandemic. In this balance, we must consider school as well, including children's right to education.

Our work has limitations. First, as the dataset is based on news available on the website, it is possible that it underestimates the real situation. Second, the cases in the database are as reliable as the newspapers that publish the news. Although the authors of the database carefully assess the news and collect several information (excluding that news with no enough information), they do not perform a formal investigation to verify the truthfulness or reliability of the news with individual schools. Third, news publishing inevitably suffers from some bias. For example, reports of cases in small towns rather than in large cities seem more frequent, possibly because the former are promptly communicated on the municipal web pages. Data about contact tracing initiated by the health authorities are missing most times, probably because news agencies prefer to focus on putting more emphasis on situations that see the emergence of an outbreak. Fourth, SARS-CoV-2 status of other family members was not assessed. However, official data on patterns of SARS-CoV-2 infection within schools and classes, and in particular according to different age groups, are still lacking in Italy, and this study represents the first nationwide analyses addressing this priority topic. Such data are needed to support policy makers and to provide baseline information for future comparisons.

In conclusion, our preliminary data suggest that, so far, a limited number of students have been diagnosed with SARS-CoV-2 infection and that intraclass transmission is rare. Active monitoring of the trend and drivers of infections among school is necessary, hopefully with open-access data on international platforms, allowing better analyses and comparisons between different settings. Importantly, understanding how different age groups can spread the infection within schools is a priority, as this would allow the development of guidelines more focused on specific school-settings. The goal of our society should be to try to respect, as much as possible, children's rights.

## Data Availability Statement

The datasets presented in this study can be found in online repositories. The names of the repository/repositories and accession number(s) can be found in the article/supplementary material.

## Ethics Statement

Ethical review and approval was not required for the study on human participants in accordance with the local legislation and institutional requirements. Written informed consent from the participants' legal guardian/next of kin was not required to participate in this study in accordance with the national legislation and the institutional requirements.

## Author Contributions

DB conceptualized the study. CD and RM extracted the data. RM made the statistical analyses. PV provided the intellectual support. All authors contributed with the last version of the manuscript.

## Conflict of Interest

The authors declare that the research was conducted in the absence of any commercial or financial relationships that could be construed as a potential conflict of interest.
